# Anthraquinones of the Roots of *Pentas micrantha*

**DOI:** 10.3390/molecules18010311

**Published:** 2012-12-27

**Authors:** Milkyas Endale, Annabel Ekberg, John Patrick Alao, Hoseah M. Akala, Albert Ndakala, Per Sunnerhagen, Máté Erdélyi, Abiy Yenesew

**Affiliations:** 1 Department of Chemistry, University of Nairobi, P.O. Box 30197-00100, Nairobi, Kenya; 2 Department of Chemistry and Molecular Biology, University of Gothenburg, SE-412 96 Gothenburg, Sweden; 3 United States Army Medical Research Unit-Kenya, MRU 64109, APO, AE 09831, USA; 4 Swedish NMR Center, University of Gothenburg, P.O. Box 465, SE-405 30 Gothenburg, Sweden

**Keywords:** anthraquinone, malaria, *Pentas micrantha*, Rubiaceae, 5,6-dihydroxylucidin-11-*O*-methyl ether

## Abstract

*Pentas micrantha* is used in the East African indigenous medicine to treat malaria. In the first investigation of this plant, the crude methanol root extract showed moderate antiplasmodial activity against the W2- (3.37 μg/mL) and D6-strains (4.00 μg/mL) of *Plasmodium falciparum* and low cytotoxicity (>450 μg/mL, MCF-7 cell line). Chromatographic separation of the extract yielded nine anthraquinones, of which 5,6-dihydroxylucidin-11-*O*-methyl ether is new. Isolation of a munjistin derivative from the genus *Pentas* is reported here for the first time. The isolated constituents were identified by NMR and mass spectrometric techniques and showed low antiplasmodial activities.

## 1. Introduction

The Rubiaceae family is an exceptional source of antimalarial herbs in East African traditional medicine [1]. 9,10-Anthraquinones, biosynthesized through the chorismate/*O*-succinylbenzoic acid pathway, are common secondary metabolites in this family [2]. Due to their diverse biological activities, 9,10-anthraquinones are receiving growing attention. They were reported to possess antiviral [3], antibacterial [4], antifungal [5], protein cleaving [6], anticancer [7], and antimalarial [8,9] properties, for example. Among the 450 genera of Rubiaceae, the genus *Pentas* became known for its rich anthraquinone and pyranonaphthoquinone content [10], which is responsible for the antiplasmodial activity of the root extracts of some of its species [11] that are regularly utilized in the Kenyan indigenous medicine for the treatment of malaria [12,13]. The urgent need for scientific evaluation of herbal medicines was demonstrated by recent studies of three morphologically similar *Pentas* species utilized in the Kenyan indigenous practice, revealing good antiplasmodial activity of the alcoholic root extract of *P. lanceolata* (Forssk.) (IC_50_ = 1.3–2.5 μg/mL) [11], the lack of activity of that of *P. bussei* (K. Krause) (IC_50_ = 49.0–49.9 μg/mL) [14] and the high cytotoxicity of the extract of the roots of *P. longiflora* (Oliv.) (IC_50_ = 0.9–1.0 μg/mL; CC_50_ = 0.8 μg/mL) [11]. As part of our ongoing efforts to evaluate the antimalarial activity of Kenyan *Pentas* species that are in use in traditional medicine we have investigated *Pentas micrantha* (Baker). In East Africa its fresh roots are chewed or its root infusion is taken against malaria [15]. Herein the very first phytochemical examination of its roots is presented.

## 2. Results and Discussion

Despite its indigenous use in malaria treatment [15], *Pentas micrantha* (Baker) has so far not been phytochemically examined. Chromatographic separation of the methanol extract provided nine compounds, of which tectoquinone (**1**), lucidin-*ω*-methyl ether (**2**), damnacanthol (**3**), rubiadin-1-methyl ether (**4**), rubiadin (**5**), damnacanthal (**7**), 5,6-dihydroxydamnacanthol (**8)**, and munjistin methyl ester (**9**) [16,17] were previously reported from other plants [11]. It should be noted that this is the first report on isolation of a munjistin derivative from the genus *Pentas*. In addition to the above quinones, the new natural product **6** was isolated as a red powder. Its HRMS (ESI) analysis indicated the molecular formula C_16_H_12_O_7_, whereas its UV-VIS absorption maxima at 216, 270, 310 and 420 nm were compatible with a 9,10-anthraquinone skeleton [18]. Its ^1^H-NMR spectrum (Table 1) displayed an aromatic singlet, H-4, and a pair of *ortho-*coupled aromatic doublets for H-7 and H-8, indicating the presence of a tri- and a disubstituted aromatic rings in the anthraquinone system. The chemical shift of the additional peaks revealed the presence of a methoxy (OCH_3_-12), an oxymethylene (CH_2_-11), and a chelated hydroxyl group (OH-1).

HMBC correlations of the phenolic proton OH-1, observable for a DMSO-*d*_6_ solution, to C-9 (Table 1) allowed its assignation to the C-1 position. The HMBC correlations of H-4 (s) to the C-10 carbonyl, C-2 and C-3 aromatic carbons confirmed that ring A is trisubstituted, and allowed its unambiguous positioning at C-4. The chemical shift of OCH_3_-12 along with its HMBC correlation to C-11 as well as the correlations of CH_2_-11 to C-1, C-2 and C-3 indicated the presence of a methoxymethyl substituent at C-2. The occurrence of a directly attached carbon substituent at the C-2 position is in agreement with the generally accepted biogenetic route of anthraquinones of the Rubiaceae family [2]. H-8 was assigned based on its HMBC correlation to C-9, which assignation also defines the position of H-7 on ring C, whereas 6- and 3-OH were assignable based on the C-3 and C-6 chemical shifts. The above spectroscopic evidence allowed the characterization of **6** as 1,3,5,6-tetrahydroxy-2-(methoxymethyl)-9,10-anthraquinone, for which the trivial name 5,6-dihydroxylucidin-11-*O*-methyl ether is proposed.

**Table 1:** The ^1^H and ^13^C-NMR data for 5,6-dihydroxylucidin-11-*O*-methyl ether (**6**) in DMSO-*d*_6_. 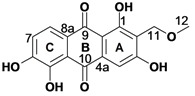

Atom	δ_H_ (I, mult, *J* in Hz)	δ_C_	HMBC
1	-	164.4	-
1a	-	109.1	-
2	-	117.4	-
3	-	163.6	-
4	7.20 (1H, s)	107.2	1a, 2, 10
4a	-	134.2	-
5	-	152.4	-
5a	-	116.2	-
6	-	152.9	-
7	7.06 (1H, d, 8.1)	120.9	5,8a
8	7.63 (1H, d, 8.1)	121.8	5a,6,9
8a	-	123.3	-
9	-	184.0	-
10	-	188.6	-
11	4.42 (2H, s)	61.7	1,2,3,12
12	3.25 (3H, s)	58.0	11
1-OH	13.92 (1H, br s)	-	1,1a,2
3-OH	-	-	-
5-OH	-	-	-
6-OH	-	-	-

As roots of herbs belonging to the genus *Pentas* and so *P. micrantha* are used in the Kenyan traditional medicine against malaria-related fever, the antiplasmodial activity and cytotoxicity of the crude methanol root extract was investigated. A promising *in vitro* antiplasmodial activity (Table 2) was observed along with low cytotoxicity. The higher antiplasmodial activity of the crude methanol extract as compared to that of its isolated constituents may be rationalized by synergistic effects [19] or by the possible loss of a highly active minor constituent throughout the isolation process. The observed weak-to-moderate antiplasmodial activity of the isolated 9,10-anthraquinones is corroborated by previous reports [20,21,22,23]. The acquired data indicates that the substitution pattern of the anthraquinone skeleton modulates the antiplasmodial activity, with compound **5** showing the highest activity. Although preliminary suggestions on the structure-activity relationship of this compound group have already been presented [20], additional studies are required for the establishment of reliable conclusions.

**Figure 1:**
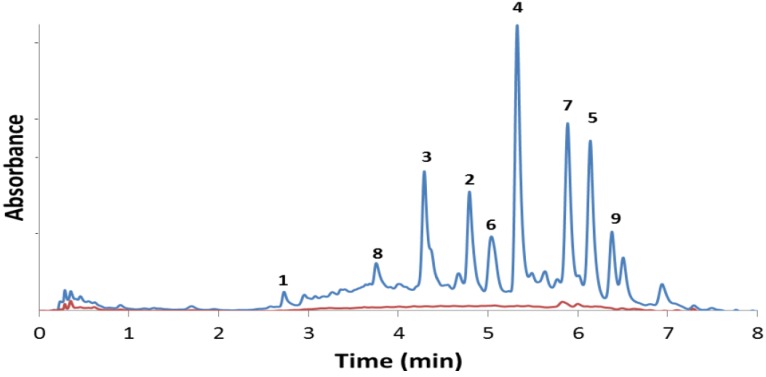
HPLC chromatograms of the methanol (blue) and aqueous (red) extracts of the roots of *Pentas micrantha*. The isolated constituents are given in Table 2. A 2.5 mL/min flow rate of acetonitrile/water eluent (0.1% formic acid, isocratic 30:70 for 2 min, gradient 30:70 to 70:30 in 5 min, isocratic 70:30 for 1 min) was used on a Gemini C-18 column (5 mm, 100 Å).

The analytical HPLC-UV chromatogram [24] of the alcoholic root extract (Figure 1) indicated that the major anthraquinone constituents were successfully isolated. Furthermore, it confirms that compounds **1**–**9** are truly present in the roots and are unaffected by the purification process [25]. Compounds **1**–**9** were also identified from the chloroform and the ethyl acetate extracts of the roots (Supporting Information, Figure S7) further confirming that neither methyl ethers **2**–**4**, **7** and **8** or methyl ester **9** are artifacts produced by the methanolic extraction process.

**Table 2:** Biological activities of the methanol root extract of *P. micrantha* and of the isolated constituents **1**–**9**. 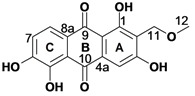

Sample	R^1^	R^2^	R^3^	R^5^	R^6^	IC_50_ (μmol/mL) ^a^		CC_50_^b^
						D6	W2	(μmol/mL)
Crude (CH_3_OH)						4.00 ± 1.86^c^	3.37 ± 0.74^c^	>450 ^c^
1	H	CH_3_	H	H	H	30.36 ± 0.01	48.56 ± 0.01	>100
**2**	OH	CH_2_OCH_3_	OH	H	H	42.54 ± 0.01	46.44 ± 0.01	>352
**3**	OCH_3_	CH_2_OH	OH	H	H	56.58 ± 0.00	110.6 ± 0.01	238
**4**	OCH_3_	CH_3_	OH	H	H	45.07 ± 0.01	70.56 ± 0.00	208
**5**	OH	CH_3_	OH	H	H	21.54 ± 0.00	31.91 ± 0.01	310
**6**	OH	CH_2_OCH_3_	OH	OH	OH	31.84 ± 0.01	35.41 ± 0.01	258
**7**	OCH_3_	CHO	OH	H	H	27.19 ± 0.00	38.58 ± 0.00	316
**8**	OCH_3_	CH_2_OH	OH	OH	OH	47.53 ± 0.01	61.17 ± 0.02	>450
**9**	OH	COOCH_3_	OH	H	H	n.d.^d^	n.d. ^d^	n.d. ^d^

^a^ IC_50_: *In vitro* activity. Data are the mean of at least 3 independent experiments; ^b^ CC_50_: cytotoxic concentration. The mean values of at least six independent experiments are given; ^c^For crude extract the data is given in μg/mL; ^d^ n.d.: not determined due to the low isolated amount (0.5 mg). As positive controls 1-isopropyl-3-(pyridin-4-ylethynyl)-1H-pyrazolo[3,4-d]pyrimidin-4-amine [26] (CC_50_ = 5.0 μmol/mL, confidence interval (95%) = 1.4–17.8 μmol/mL), chloroquine (IC_50_ = 14.68 ± 2.41 nmol/mL (D6); IC_50_ = 522.4 ± 84.2 nmol/mL (W2)) and mefloquine (IC_50_ = 87.61 ± 40.46 nmol/mL (D6); IC_50_ = 6.65 ± 1.18 nmol/mL (W2)) were used. The bioactivity of compounds **1**–**5** and **7**–**8** was previously published in reference [11].

A small quantity of damnacanthal (**7**) was identified by HPLC-UV (Figure 1, red curve) and confirmed by NMR in the aqueous root extract, which observation reveals that the typical indigenous use of *P. micrantha*—the chewing of its roots or taking of its aqueous (or alcoholic) root infusion—may have some antiplasmodial effect [15]. However, the 9,10-anthraquinone content, often directly associated with antiplasmodial activity of *Pentas* extracts, is significantly lower in the aqueous as compared to the alcoholic extract (Figure 1). It should also be noted that the alcoholic extract does not show significant cytotoxicity (Table 2).

## 3. Experimental

### 3.1. General Procedures

Column chromatography was performed on oxalic acid impregnated silica gel, following a previously described procedure [17]. TLC was carried out on precoated silica gel 60 F_254_ (Merck) plates. For structure elucidation gCOSY, gTCOSY, gNOESY, gHSQC and gHMBC NMR spectra were acquired on Varian 800, 500 and 400 MHz spectrometers. LC-MS (ESI) was performed on a Perkin Elmer PE SCIEX API 150 EX instrument equipped with Turbolon spray ion source and a Gemini 5 mm C-18 110 Ǻ HPLC column using a water-acetonitrile gradient (80:20 to 20:80). High-resolution mass spectral analysis (Q-TOF-MS) was performed at Stenhagen Analyslab AB, Gothenburg, Sweden. Preparative HPLC was run on a Waters 600E HPLC system using the Chromulan software (Pikron Ltd., Praha, Czech Republic), a Kromasil C-8 250 × 25 mm C-8 column and water-acetonitrile eluent mixture.

The crude methanol and aqueous root extracts were analyzed using analytical HPLC applying a gradient of water-acetonitrile (0.1% formic acid) with 2.5 mL/min flow rate on a Gemini C-18 column (5 mm, 110 Å) using a LaChrom Elite HPLC system. An isocratic CH_3_CN:H_2_O (30:70) flow for 2 min was followed by the CH_3_CN:H_2_O gradient of 30:70 to 70:30 in 5 min, and isocratic 70:30 for 1 min. The presence of the purified constituents **1**–**8** in the crude extracts was confirmed by comparison of retention times.

### 3.2. Plant Material

*P. micrantha*, collected at the coastal region of Kenya in July 2010, was identified by P. Chalo Mutiso at the Herbarium, School of Biological Sciences (SBS), University of Nairobi (UoN), where a voucher specimen was deposited (nr. MEA-2010/004).

### 3.3. Extraction and Isolation

The air-dried roots (0.8 kg, 5 mm mesh) were pulverized and extracted with CH_2_Cl_2_:CH_3_OH (1:1, 4 times 1.5 L) for 24 h at room temperature. The extract was concentrated under vacuum using a rotary evaporator to yield a brownish crude extract (47 g, 5.87%). A portion of the crude extract (45 g) was subjected to column chromatography (80 cm length and 8 cm diameter, 400 g oxalic acid impregnated silica gel [11]) using an increasing gradient of EtOAc in *n*-hexane and 250 fractions, 200 mL each, were collected. Fractions 10–12 (1% EtOAc in *n*-hexane) were combined and purified on Sephadex LH-20 (SLH-20) to give tectoquinone [11] (**1**, 15 mg). Following re-purification on SLH-20 (eluent, CH_2_Cl_2_:CH_3_OH; 1:1), fractions 15–20 (2% EtOAc) yielded lucidin-*ω*-methyl ether [17] (**2**, 80 mg). Fractions 63–64 (5% EtOAc) were combined and purified using column chromatography (increasing gradient of EtOAc in *n*-hexane) followed by SLH-20 (eluent, CH_2_Cl_2_:CH_3_OH; 1:1) to give damnacanthol [17] (**3**, 52 mg). Rubiadin-1-methyl ether [17] (**4**, 25 mg) was obtained from fractions 75–79 (7% EtOAc in *n*-hexane), following repurification on SLH-20 (CH_2_Cl_2_: CH_3_OH; 1:1). Fractions 100–103 (10% EtOAc) gave rubiadin [17] (**5**, 25 mg). Fractions 115–118 (12% EtOAc in *n*-hexane) were combined and subjected to column chromatography (increasing gradient of EtOAc in *n*-hexane) to yield 5,6-dihydroxylucidin-11-*O*-methyl ether (**6**, 30 mg). Fractions 131–135 (18% ethyl acetate in *n*-hexane) were re-purified using column chromatography (increasing gradient of EtOAc in *n*-hexane) to give damnacanthal [17] (**7**, 30 mg). Fractions 150–155 (30% EtOAc in *n*-hexane) gave 5,6-dihydroxydamnacanthol [17] (**8**, 25 mg) following column chromatography (increasing gradient of EtOAc in *n*-hexane).

The root residue was further extracted with CH_3_OH (100%), three times (2.5 L) for 24 h and following filtration the extract was concentrated on a rotary evaporator to yield a brownish solid extract (41 g, 5.1%). Using column chromatography (80 cm length and 80 mm diameter, 400 g oxalic acid impregnated silica gel; increasing gradient of CH_3_OH in CH_2_Cl_2_) 40 g of the crude extract was purified, collecting 200 fractions of 200 mL each. Anthraquinones **1**–**8** were reisolated from the methanol extract following the above described chromatographic methods. HPLC separation (Waters 600E using the software Chromulan, Pikron Ltd.) of the crude extract (11.8 g root, 200 mL CH_3_OH) on a preparative C-8 reversed phase column (X-Terra^TM^ MS C-8, 5 μm, 19 × 50 mm) with a water-methanol gradient of 30:70 to 80:20 for 30 min, followed by 10 min at 80:20 provided munjistin methylester [16,17] (**9**, 0.5 mg) in addition to compounds **1**–**8**.

The crude CH_2_Cl_2_:CH_3_OH (1:1) and CH_3_OH extracts were analyzed using analytical RP-HPLC applying a gradient of acetonitrile/water (0.1% formic acid) with 2.5 mL/min flow rate on a Gemini C-18 column (5 mm, 100 Å) using a LaChrom Elite HPLC system. An isocratic CH_3_CN:H_2_O (30:70) flow for 2 min was followed by the CH_3_CN:H_2_O gradient of 30:70 to 70:30 in 5 min. The presence of the purified constituents **1**–**9** in the crude extracts was confirmed by comparison of retention times.

### 3.4. Drugs

The reference antimalarial drugs chloroquine (Sigma-Aldrich) and mefloquine (Sigma Aldrich) that have well-documented IC_50_ values were tested alongside the crude root extract and the isolated anthraquinones. For cytotoxicity (CC_50_) assay 1-isopropyl-3-(pyridin-4-ylethynyl)-1H-pyrazolo[3,4-d]pyrimidin-4-amine [26] was used as reference substance.

### 3.5. Drug Susceptibility Testing

Sierra Leone D6 chloroquine-sensitive and the Indochina W2 chloroquine-resistant *Plasmodium falciparum* strains (donated by the Walter Reed Army Institute for Research [WRAIR], 503 Robert Grant Avenue, Silver Spring, MD 20910, USA) were maintained in continuous culture to attain replication robustness prior to assays. Drug susceptibility was tested by the Malaria SYBR Green I-based *in vitro* assay technique [15,17].

### 3.6. Cytotoxicity Assay

MCF-7 human breast cancer cells were cultured in Dulbecco’s modified eagle medium (DMEM) supplemented with 10% (v/v) fetal bovine serum, 2 mM L-glutamine, 100 units/mL penicillin and 100 µg/mL streptomycin at 37 °C in humidified 5% CO_2_. For cytotoxicity assays, cells were seeded in 96-well plates at optimal cell density to ensure exponential growth for the duration of the assay. After a 24-h preincubation growth, the medium was replaced with experimental medium containing the appropriate drug concentrations or vehicle controls (0.1% or 1.0% v/v DMSO). After 48 h incubation, cell viability was measured using PrestoBlue™ Cell Viability Reagent (Invitrogen Ab, Lidingö, Sweden) according to the manufacturer’s instructions. Absorbance was measured at 570 nm with 600 nm as a reference wavelength. Results were expressed as the mean ± standard error for six replicates as a percentage of vehicle control (taken as 100%). Experiments were performed independently at least six times. Statistical analyses were performed using a two-tailed Student’s t-test. *p* < 0.05 was considered to be statistically significant.

### 3.7. Spectral Data

*Tectoquinone* (**1**)*.* Orange solid. ^1^H-NMR (200 MHz, CDCl_3_, 25 °C) *δ* 8.39 (d, *J* = 5.8 Hz, H-8), 8.37 (d, *J* = 5.8 Hz, 1H, H-5), 8.29 (d, *J* = 8.1 Hz, H-4), 8.19 (d, *J* = 0.4 Hz, H-1), 7.87 (d, *J* = 5.8 Hz, H-7), 7.85 (d, *J* = 5.8 Hz, 1H, H-6), 7.80 (dd, *J* = 0.4, 8.1 Hz, H-3), 2.62 (s, 3H, CH_3_-11). ^13^C-NMR (50 MHz, CDCl_3_, 25 °C) *δ* 183.8 (C-9), 183.8 (C-9), 183.3 (C-10), 145.6 (C-2), 135.3 (C-3), 134.4 (C-6), 134.2 (C-7), 133.9 (C-1a), 133.88 (C-4a), 133.7 (C-5a), 131.6 (C-8a), 127.8 (C-1), 127.7 (C-4), 127.5 (C-5 and C-8), 22.2 (C-11). *m/z* (ESI-MS, 30 eV) 223.3 [M+H]^+^ [11].

*Lucidine-ω-methyl ether* (**2**)*.* Orange solid. UV (CH_3_OH) λ_max_ nm 230, 280, 410 nm. ^1^H-NMR (DMSO-*d_6_*, 799.89 MHz, 25 °C) δ 13.31 (s, 1H, OH), 11.5 (br s, 1H, OH), 8.25 (ddd, *J* = 0.8, 1.8, 7.3 Hz, 1H, H-5), 8.17 (ddd, *J* = 0.8, 1.7, 7.3 Hz, 1H, H-8), 7.81 (ddd, *J* = 1.8, 7.3, 7.3 Hz, 1H, H-7), 7.78 (ddd, *J* = 1.8, 7.3, 7.3 Hz, 1H, H-6), 7.33 (s, 1H, H-4), 4.54 (s, 2H, CH_2_-11), 3.43 (s, 3H, CH_3_-12). ^13^C-NMR (DMSO-*d_6_*, 199.95 MHz, 25 °C) δ 186.9 (C-10), 182.4 (C-9), 165.0 (C-3), 164.5 (C-1), 135.5 (C-4a), 135.3 (C-5a), 134.7 (C-8a), 133.8 (C-7), 133.6 (C-6), 127.6 (C-5), 127.2 (C-8), 117.5 (C-2), 109.8 (C-1a), 108.5 (C-4), 62.0 (C-11), 58.2 (C-12). *m/z* (ESI-MS, 30 eV) 285.3 [M+H]^+^, 265.3, 256.8, 247.4 [11].

*Damnacanthol* (**3**). Orange solid. UV (CH_3_OH) λ_max_ 225, 270, 365 nm. ^1^H-NMR (DMSO-*d_6_*, 799.89 MHz, 25 °C) δ 11.17 (br s, 1H, OH), 8.15 (dd, *J* = 1.40, 7.70 Hz, 1H, H-8), 8.10 (dd, *J* = 1.40, 7.70 Hz, 1H, H-5), 7.89 (ddd, *J* = 1.40, 7.70, 7.70 Hz, 1H, H-7), 7.83 (ddd, *J* = 1.40, 7.70, 7.70 Hz, 1H, H-6), 7.51 (s, 1H, H-4), 4.56 (s, 2H, CH_2_-11), 3.85 (s, 3H, CH_3_-12). ^13^C-NMR (DMSO-*d_6_*, 199.95 MHz, 25 °C) δ 185.6 (C-10), 183.1 (C-9), 165.2 (C-3), 164.9 (C-1), 138.9 (C-4a), 137.7 (C-7), 136.5 (C-6), 136.2 (C-8a), 135.3 (C-5a), 132.0 (C-2), 129.9 (C-8), 129.2 (C-5), 121.0 (C-1a), 112.9 (C-4), 65.5 (C-12), 56.2 (C-11). *m/z* (ESI-MS, 30 eV) 307.4 [M+Na]+, 285.4 [M+H]^+^, 267.3 [11].

*Rubiadin-1-methyl ether* (**4**). Orange solid. UV (CH_3_OH) λ_max_ 225, 265, 280, 360 nm. ^1^H-NMR (DMSO-*d_6_*, 500 MHz, 25 °C) δ 8.11 (d, *J* = 5.0 Hz, 1H, H-8), 8.06 (d, *J* = 5.0 Hz, 1H, H-5), 7.85 (dd, *J* = 5.0, 7.2 Hz, 1H, H-7), 7.79 (dd, *J* = 5.0, 7.2 Hz, 1H, H-6), 7.46 (s, 1H, H-4), 3.76 (s, 2H, H-12), 2.13 (s, 3H, H-12). ^13^C-NMR (DMSO-*d_6_*, 500 MHz, 25 °C) δ 185.5 (C-9), 183.1 (C-10), 164.6 (C-3), 163.7 (C-1), 137.6 (C-7), 136.8 (C-4a), 136.3 (C-6), 135.2 (C-5a), 135.1 (C-8a), 129.2 (C-2), 129.8 (C-8), 129.3 (C-5), 120.9 (C-1a), 112.1 (C-4), 63.7 (C-12), 12.1 (C-11). *m/z* (ESI-MS, 30 eV) 307.4 [M+K]^+^, 291.3 [M+Na]^+^, 269.6 [M+H]^+^ [11].

*Rubiadin* (**5**) Yellow solid. UV (CH_3_OH) λ_max_ 225, 240, 270, 280, 405 nm. ^1^H-NMR (DMSO-*d_6_*, 799.89 MHz, 25 °C) δ 13.8 (s, OH), 11.1 (br s, 1-OH), 8.06 (dd, *J* = 1.9, 7.9 Hz, 1H, H-8), 8.02 (dd, *J* = 1.6, 7.0 Hz, 1H, H-5), 7.90 (ddd, *J* = 1.9, 7.0, 7.9 Hz, H-7), 7.82 (ddd, *J* = 1.6, 7.0, 7.0 Hz, 1H, H-6), 7.11 (s, 1H, H-4), 1.97 (s, 3H, CH_3_-11). ^13^C-NMR (DMSO-*d_6_*, 199.95 MHz, 25 °C) δ 186.0 (C-9), 181.5 (C-10), 162.7 (C-3), 162.1 (C-1), 134.3 (C-6), 134.2 (C-7), 132.8 (C-5a), 132.7 (C-8a), 131.5 (C-4a), 126.5 (C-8), 126.2 (C-5), 117.2 (C-2), 108.8 (C-1a), 107.3 (C-4), 9.5 (CH_3_-11). *m/z* (ESI-MS, 30 eV) 255.6 [M+H]^+^, 239.2, 212.3, 171.5 [11].

*5,6-Dihydroxylucidin-11-O-methyl ether* (**6**). Red solid. UV (CH_3_OH) λ_max_ 216, 270, 310 and 420 nm, ^1^H-NMR and ^13^C-NMR (Table 1). HRMS (ESI): *m/z* 315.0571 [M−H]^−^, calcd. for C_16_H_11_O_7_: 315.0524. Spectra are shown in the ESI.

*Damnacanthal* (**7**). Orange solid. UV (CH_3_OH) λ_max_ 230, 280, 380 nm. ^1^H-NMR (DMSO-*d_6_*, 799.89 MHz, 25 °C) δ 12.10 (br s, 1H, OH), 10.40 (s, 1H, H-10), 8.18 (d, *J* = 7.9 Hz, 1H, H-8), 8.14 (d, *J* = 7.9 Hz, 1H, H-5), 7.94 (dd, *J* = 7.9, 7.9 Hz, 1H, H-7), 7.88 (dd, *J* = 7.9, 7.9 Hz, 1H, H-6), 7.49 (s, 1H, H-4), 3.97 (s, 3H, CH_3_-12). ^13^C-NMR (DMSO-*d_6_*, 199.95 MHz, 25 °C) δ 196.0 (C-11), 185.1 (C-10), 182.9 (C-9), 168.3 (C-1), 167.7 (C-3), 143.4 (C-4a), 138.1 (C-7), 137.8 (C-8a), 136.7 (C-6), 135.6 (C-5a), 129.9 (C-8), 129.5 (C-5), 123.7 (C-2), 120.8 (C-1a), 113.9 (C-4), 66.8 (CH_3_-12). *m/z* (ESI-MS, 30 eV) 305.3 [M+Na]^+^, 283.3 [M+H]^+^ [11].

*5-6-Dihydroxydamnacanthol* (**8**). Red solid. UV (CH_3_OH) λ_max_ 424, 308, 274, 218 nm. ^1^H-NMR (DMSO-*d_6_*, 799.89 MHz, 25 °C) δ 12.4 (br s, 1H, OH), 7.54 (d, *J* = 8.2 Hz, 1H, H-8), 7.52 (s, 1H, H-4), 7.18 (d, *J* = 8.2 Hz, 1H, H-7), 4.92 (br s, 1H, OH), 4.52 (s, 2H, CH_2_-11), 3.79 (s, 3H, CH_3_-12). ^13^C-NMR (DMSO-*d_6_*, 199.95 MHz, 25 °C) δ 189.2 (C-10), 179.4 (C-9), 162.4 (C-1), 162.3 (C-3), 151.8 (C-6), 150.7 (C-5), 135.8 (C-4a), 130.1 (C-8a), 126.1 (C-2), 121.8 (C-8), 121.1 (C-7), 118.8 (C-5a), 116.4 (C-1a), 110.3 (C-4), 63.0 (CH_3_-12), 52.8 (CH_2_-11). *m/z* (ESI-MS, 30 eV) 317.1 [M+H]^+^, 299.6 [M−18]^+^ [11].

*Munjistin methylester* (**9**). Yellow solid. ^1^H-NMR (DMSO-*d_6_*, 799.89 MHz, 25 °C) δ 13.1 (br s, 1H, OH), 8.11 (d, *J* = 7.5 Hz, 1H, H-8), 8.05 (d, *J* = 7.7 Hz, 1H, H-5), 7.83 (dd, *J* = 7.5, 7.7 Hz, 1H, H-7), 7.72 (dd, *J* = 7.5, 7.7 Hz, 1H, H-6), 6.53 (s, 1H, H-4), 3.66 (s, 3H, CH_3_-12). ^13^C-NMR (DMSO-*d_6_*, 199.95 MHz, 25 °C) δ 184.1 (C-10), 178.5 (C-9), 167.8 (C-11), 165.6 (C-3), 164.0 (C-1), 135.2 (C-8a), 134.3 (C-7), 132.7 (C-5a), 132.3 (C-6), 126.4 (C-5), 125.5 (C-8), 117.8 (C-4), 116.4 (C-1a), 101.0 (C-2), 51.1 (CH_3_-12). *m/z* (ESI-MS, 30 eV) 283.7 [M+H]^+^, 239.1[M−45]^+^ [17].

## 4. Conclusions

Several *Pentas* species are in indigenous use in the East African traditional medicine, however, without taking the likely variation of the chemical constituents between the species or subspecies in consideration. The anthraquinone content of the roots of *P. lanceolata* [11] and *P. micrantha* shows a high degree of similarity (quinones **1**–**5**, **7**–**8**) suggesting their close genetic relationship. On the contrary, the major secondary metabolites of *P. longiflora* and *P. bussei* are pyranonaphthoquinones and benzoquinones, respectively, without any 9,10-anthraquinone having been so far reported [11,14]. Whereas anthraquinones show moderate antiplasmodial activity [11], the benzoquinones are inactive against Plasmodium [14] and the pyranonaphthoquinone’s cytotoxicity prevents their antiplasmodial application [11]. The variation of biochemical routes between the species of genus *Pentas* along with the greatly different antiplasmodial activities of their secondary metabolites motivates further scientific analysis of this genus. In addition, it reveals the need for outreach programs to improve the public awareness of the differences between the toxicities and the antiplasmodial activities of the root extracts of the taxa of genus *Pentas*.

## Supplementary Materials

Supplementary materials can be accessed at: http://www.mdpi.com/1420-3049/18/1/311/s1.

## Supplementary Files

Supplementary File 1 Supplementary File (PDF, 494 KB)

## References

[1] Njoroge G.N., Bussmann R.W. (2006). Diversity and utilization of antimalarial ethnophytotherapeutic remedies among the Kikuyus (Central Kenya). J. Ethnobiol. Ethnomed..

[2] Han Y.S., van der Heijden R., Verpoorte R. (2001). Biosynthesis of anthraquinones in cell cultures of the Rubiaceae. Plant. Cell Tiss. Org..

[3] Koyama J., Nisino Y., Morita I., Kobayashi N., Osakai T., Tokuda H. (2008). Correlation between reduction potentials and inhibitions of Epstein-Barr virus activation by anthraquinone derivatives. Bioorg. Med. Chem. Lett..

[4] Chan K.Y., Zhang J., Chang C.W. (2011). Mode of action investigation for the antibacterial cationic anthraquinone analogs. Bioorg. Med. Chem. Lett..

[5] Singh D.N., Verma N., Raghuwanshi S., Shukla P.K., Kulshreshtha D.K. (2006). Antifungal anthraquinones from Saprosma fragrans. Bioorg. Med. Chem. Lett..

[6] Suzuki A., Hasegawa M., Ishii M., Matsumura S., Toshima K. (2005). Anthraquinone derivatives as a new family of protein photocleavers. Bioorg. Med. Chem. Lett..

[7] Wang S., Wang Q., Wang Y., Liu L., Weng X., Li G., Zhang X., Zhou X. (2008). Novel anthraquinone derivatives: Synthesis via click chemistry approach and their induction of apoptosis in BGC gastric cancer cells via reactive oxygen species (ROS)-dependent mitochondrial pathway. Bioorg. Med. Chem. Lett..

[8] Winter R.W., Cornell K.A., Johnson L.L., Isabelle L.M., Hinrichs D.J., Riscoe M.K. (1995). Hydroxy-Anthraquinones as Antimalarial Agents. Bioorg. Med. Chem. Lett..

[9] Abu el Heiga L.A., Katzhendler J., Gean K.F., Bachrach U. (1990). Antimalarial activity of substituted anthraquinones. Biochem. Pharmacol..

[10] El-Hady S., Bukuru J., Kesteleyn B., van Puyvelde L., van T.N., de Kimpe N. (2002). New pyranonaphthoquinone and pyranonaphthohydroquinone from the roots of Pentas longiflora. J. Nat. Prod..

[11] Endale M., Alao J.P., Akala H.M., Rono N.K., Eyase F.L., Derese S., Ndakala A., Mbugua M., Walsh D.S., Sunnerhagen P. (2012). Antiplasmodial Quinones from Pentas longiflora and Pentas lanceolata. Planta Med..

[12] Muthaura C.N., Rukunga G.M., Chhabra S.C., Mungai G.M., Njagi E.N. (2007). Traditional antimalarial phytotherapy remedies used by the Kwale community of the Kenyan Coast. J. Ethnopharmacol..

[13] Wanyoike G.N., Chhabra S.C., Lang’at-Thoruwa C.C., Omar S.A. (2004). Brine shrimp toxicity and antiplasmodial activity of five Kenyan medicinal plants. J. Ethnopharmacol..

[14] Endale M., Ekberg A., Akala H., Alao J.P., Sunnerhagen P., Yenesew A., Erdelyi M. (2012). Busseihydroquinines A–D from the Roots of Pentas bussei. J. Nat. Prod..

[15] Kokowaro J.O. (2010). Medicinal Plants of East Africa.

[16] Wu T.S., Lin D.M., Shi L.S., Damu A.G., Kuo P.C., Kuo Y.H. (2003). Cytotoxic anthraquinones from the stems of *Rubia wallichiana* Decne. Chem. Pharm. Bull..

[17] Kawasaki Y., Goda Y., Yoshihira K. (1992). The mutagenic constituents of Rubia tinctorum. Chem. Pharm. Bull..

[18] Scott A.I. (1964). Interpretation of the Ultraviolet Spectra of Natural Products.

[19] Jia J., Zhu F., Ma X., Cao Z., Li Y., Chen Y.Z. (2009). Mechanisms of drug combinations: Interaction and network perspectives. Nat. Rev. Drug Discov..

[20] Osman C.P., Ismail N.H., Ahmad R., Ahmat N., Awang K., Jaafar F.M. (2010). Anthraquinones with antiplasmodial activity from the roots of *Rennellia elliptica* Korth. (Rubiaceae). Molecules.

[21] Koumaglo K., Gbeassor M., Nikabu O., de Souza C., Werner W. (1992). Effects of three compounds extracted from *Morinda lucida* on *Plasmodium falciparum*. Planta Med..

[22] Fotie J. (2006). Quinones and Malaria. Antiinflamm.Antiallergy Agents Med. Chem..

[23] Onegi B., Kraft C., Kohler I., Freund M., Jenett-Siems K., Siems K., Beyer G., Melzig M.F., Bienzle U., Eich E. (2002). Antiplasmodial activity of naphthoquinones and one anthraquinone from *Stereospermum kunthianum*. Phytochemistry.

[24] Locatelli M. (2011). Anthraquinones: Analytical techniques as a novel tool to investigate on the triggering of biological targets. Curr. Drug Targets.

[25] Shamma M., Rahimizadeh M. (1986). The Identity of Chileninone with Berberrubine the Problem of True Natural-Products Vs Artifacts of Isolation. J. Nat. Prod..

[26] Diner P., Alao J.P., Soderlund J., Sunnerhagen P., Grotli M. (2012). Preparation of 3-substituted-1-isopropyl-1H-pyrazolo[3,4-d]pyrimidin-4-amines as RET kinase inhibitors. J. Med. Chem..

