# Supplementary material for: Anthraquinones of the Roots of Pentas micrantha
Source: Molecules. 2012 Dec 27;18(1):311–21. doi: 10.3390/molecules18010311 (PMC6270246; doi:10.3390/molecules18010311)

Supporting Information

Figuer S1. ^{1}H-NMR spectrum of 5,6-dihydroxylucidin-11-*O*-methyl ether (**6**), DMSO-d_6, 800 MHz.

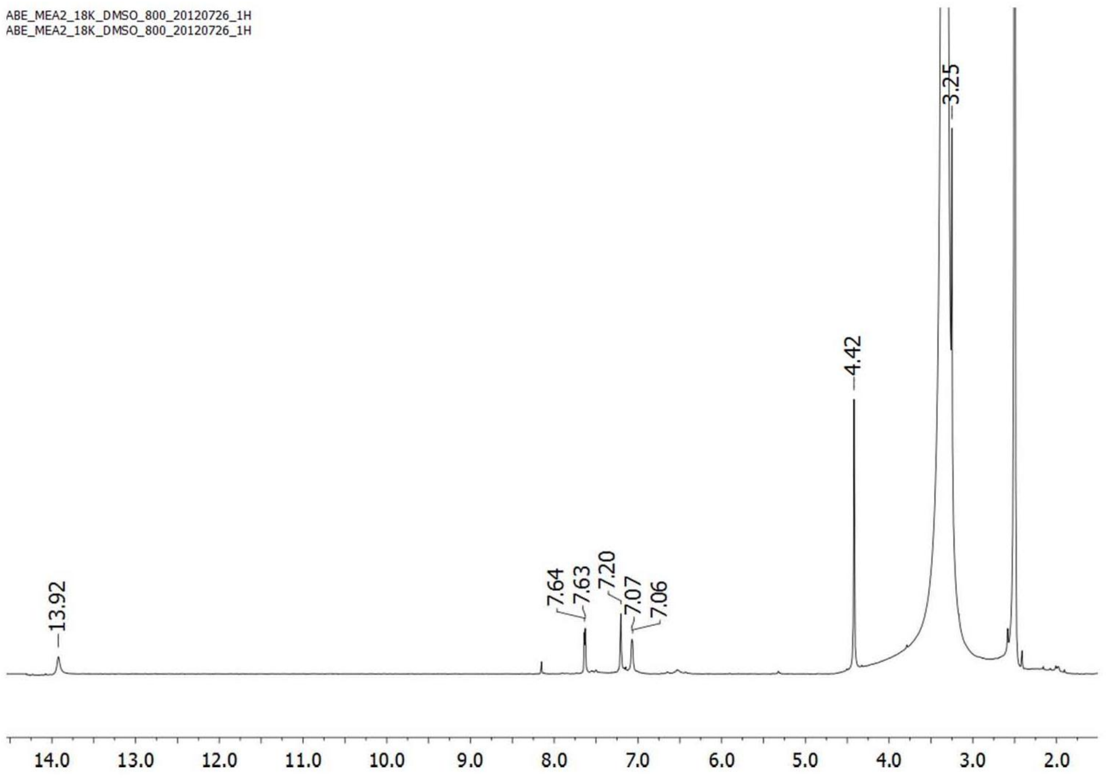

Figuer S2. ^{13}C-NMR spectrum of 5,6-dihydroxylucidin-11-*O*-methyl ether (**6**), DMSO-d_6, 150.84 MHz.

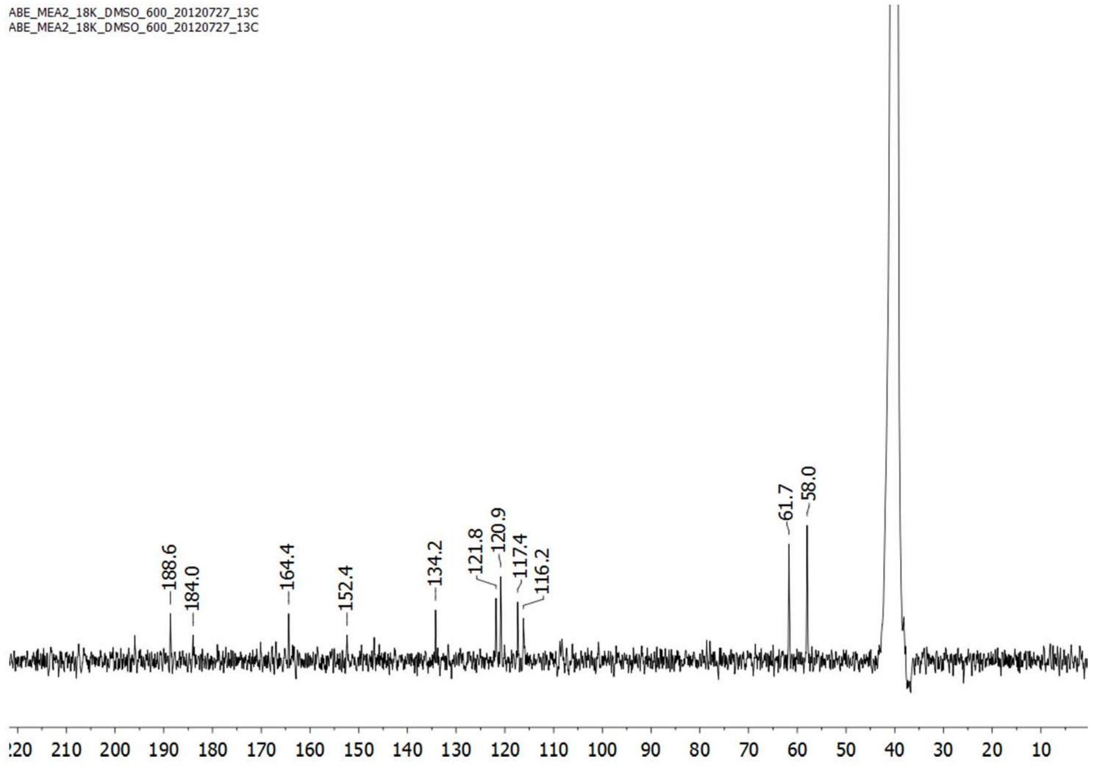

Note: The chemicals shifts of some of the quaternary carbons were derived from the ^{13}C-NMR spectrum of a sample having 80% purity and were confirmed by gHMBC (Figure S5).

Figure S3. COSY spectrum of 5,6-dihydroxylucidin-11-*O*-methyl ether (**6**), DMSO-d_6, 800 MHz.

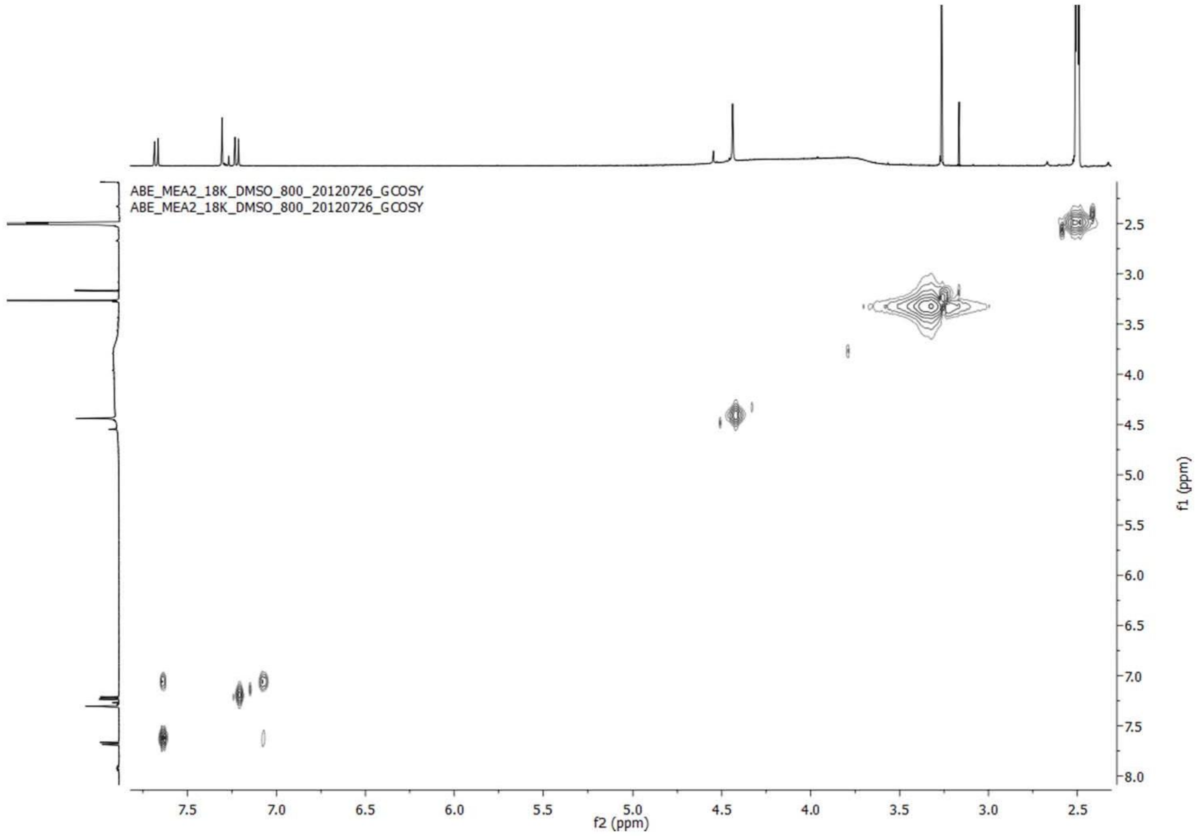

Figure S4. gHSQC Spectrum of 5,6-dihydroxylucidin-11-*O*-methyl ether (**6**), DMSO-d_6, 800 MHz.

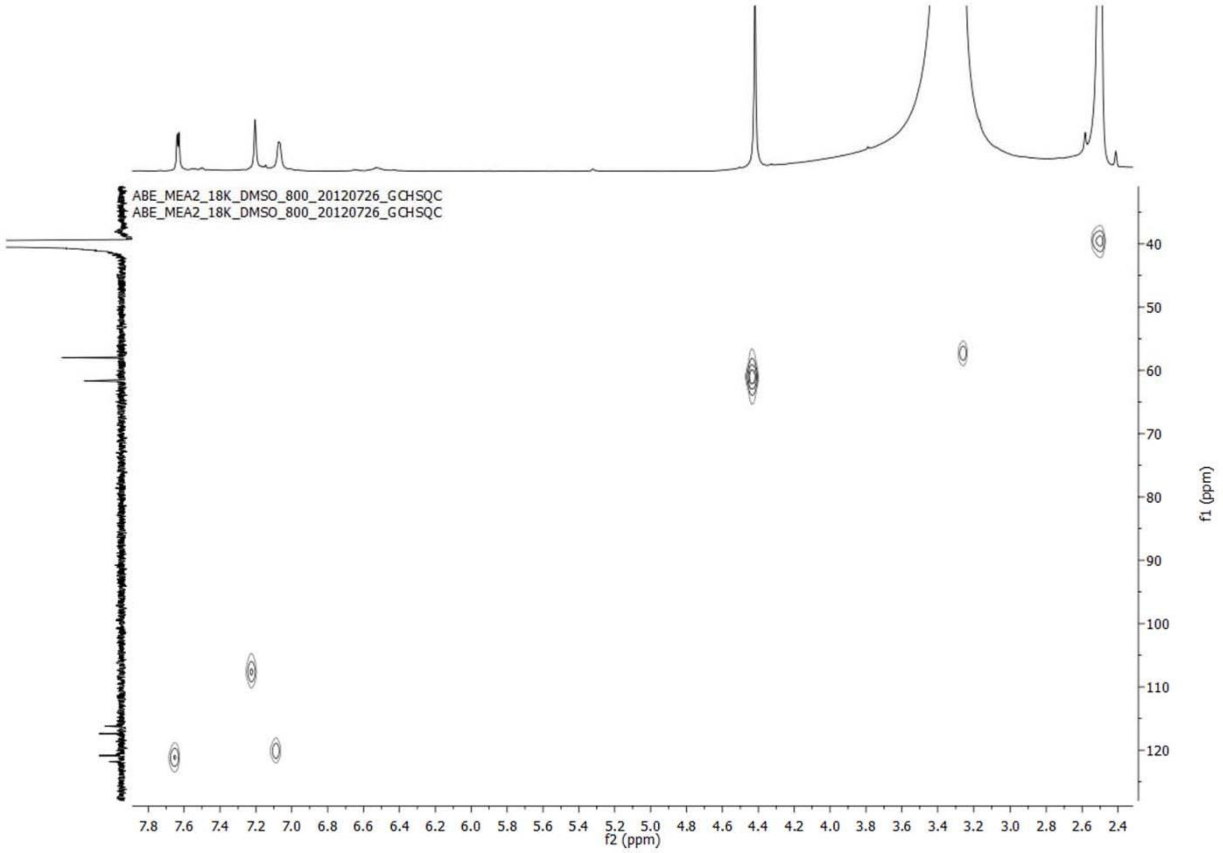

Figure S5. gHMBC Spectrum of 5,6-dihydroxylucidin-11-*O*-methyl ether (**6**), DMSO-d_6, 800 MHz.

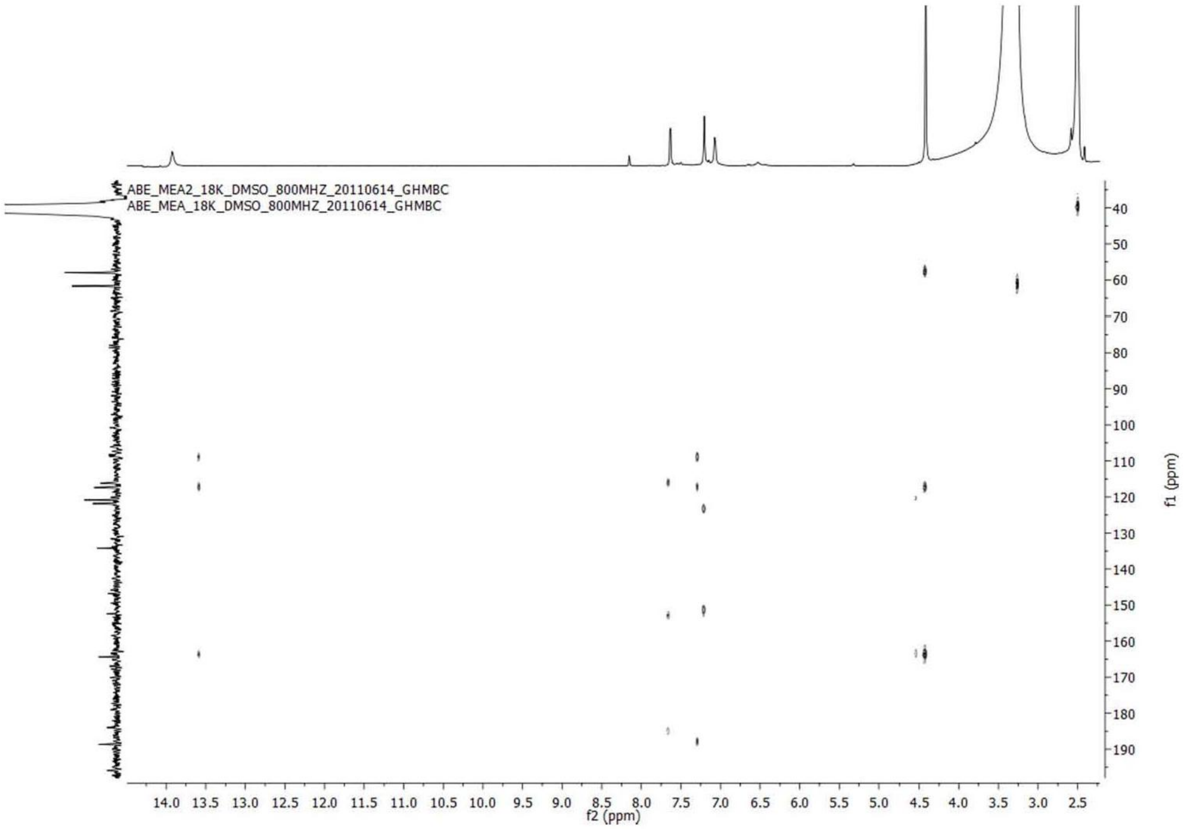

Figure S6. HRMS (ESI) spectrum of 5,6-dihydroxylucidin-11-*O*-methyl ether (**6**). ESI(+) detection with TOF scan with 100-1500 Da detection limit and 2 scan/sec detection rate. Extern calibration was applied. The sample was dissolved in acetonitrile.

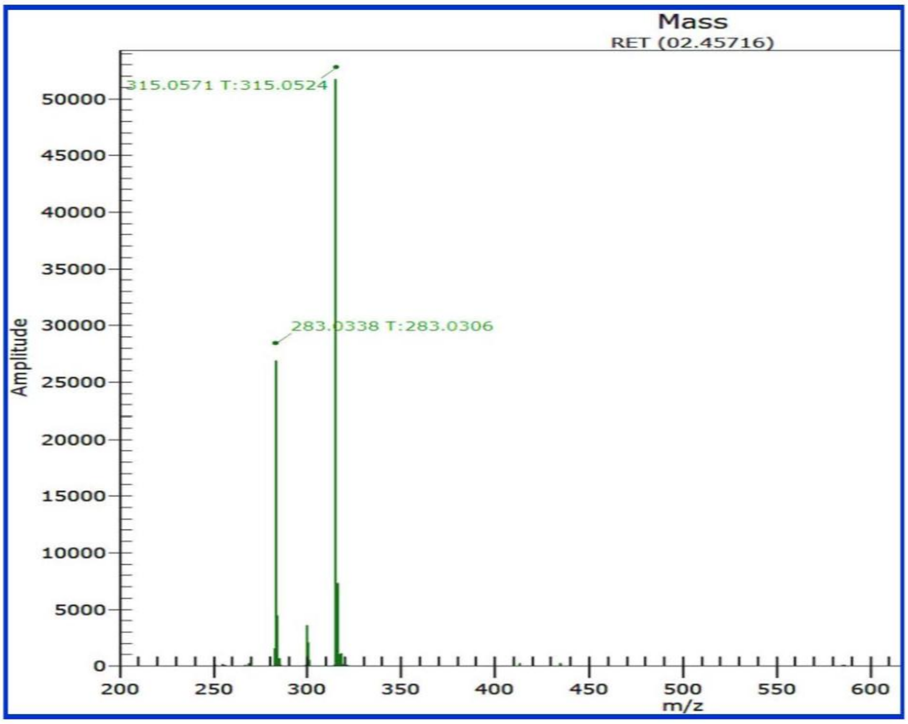

Figure S7. The HPLC chromatograms of the crude methanol (blue), chloroform (red) and ethyl acetate (green) root extracts. A water-acetonitrile (0.1% formic acid) gradient with 2.5 mL/min flow rate on a Gemini C-18 column (5 mm, 110 Å) was used. Isocratic CH3CN:H2O (30:70) flow for 2 min was followed by a CH3CN:H2O gradient of 30:70 to 70:30 in 5 minutes, and isocratic 70:30 for 1 min. Observation of compounds **1**–**9** in each extract confirms that they are not extraction artifacts resulting from methylation by methanol, for example.

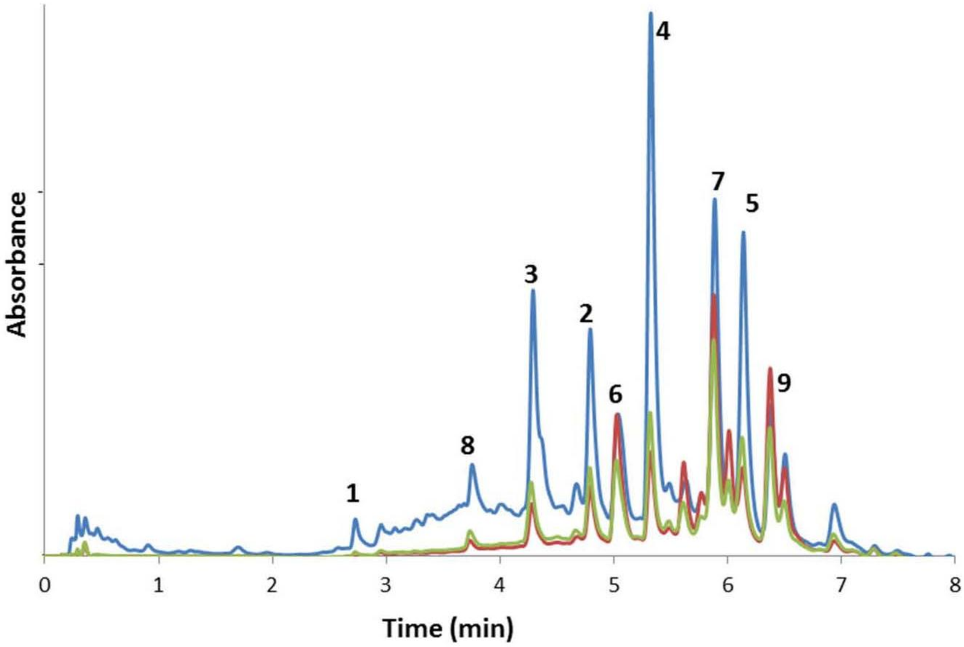

Supplement: Supplementary File 1 — Supplementary File (PDF, 494 KB) [file molecules-18-00311-s001.pdf]
